# Supplementary material for: Modeling glioblastoma heterogeneity as a dynamic network of cell states
Source: Mol Syst Biol. 2021 Sep 16;17(9):e10105. doi: 10.15252/msb.202010105 (PMC8444284; doi:10.15252/msb.202010105)
Supplement: Supplementary file 6 — Source Data for Figure 5 [file MSB-17-e10105-s004.zip › Figure5A_sourcedata/GSEA_3017/nosigdb_stateD.GseaPreranked.1622795010397/RICHARDS_NOWAKOWSKI_SCIENCE_2017_NIN2_UPREG.html]

Details for gene set RICHARDS\_NOWAKOWSKI\_SCIENCE\_2017\_NIN2\_UPREG[GSEA]

|  || Dataset | state23017 |
| Phenotype | NoPhenotypeAvailable |
| Upregulated in class | na\_pos |
| GeneSet | RICHARDS\_NOWAKOWSKI\_SCIENCE\_2017\_NIN2\_UPREG |
| Enrichment Score (ES) | 0.17748673 |
| Normalized Enrichment Score (NES) | 1.8496721 |
| Nominal p-value | 0.0 |
| FDR q-value | 0.053953294 |
| FWER p-Value | 0.074 |
Table: GSEA Results Summary

  

Fig 1: Enrichment plot: RICHARDS\_NOWAKOWSKI\_SCIENCE\_2017\_NIN2\_UPREG      
 Profile of the Running ES Score & Positions of GeneSet Members on the Rank Ordered List

  

| PROBE | GENE SYMBOL | GENE\_TITLE | RANK IN GENE LIST | RANK METRIC SCORE | RUNNING ES | CORE ENRICHMENT || 1 | SOX11 |  |  | 6 | 0.578 | 0.0170 | Yes |
| 2 | RPL10 |  |  | 53 | 0.344 | -0.0070 | Yes |
| 3 | RPL26 |  |  | 61 | 0.336 | 0.0001 | Yes |
| 4 | FAM107B |  |  | 63 | 0.329 | 0.0117 | Yes |
| 5 | PPA1 |  |  | 64 | 0.328 | 0.0241 | Yes |
| 6 | SNHG5 |  |  | 66 | 0.323 | 0.0355 | Yes |
| 7 | RPL18A |  |  | 73 | 0.314 | 0.0425 | Yes |
| 8 | NDUFB8 |  |  | 81 | 0.308 | 0.0485 | Yes |
| 9 | CALM3 |  |  | 82 | 0.308 | 0.0602 | Yes |
| 10 | RPL18 |  |  | 87 | 0.302 | 0.0683 | Yes |
| 11 | ZFAS1 |  |  | 88 | 0.301 | 0.0797 | Yes |
| 12 | SOX4 |  |  | 91 | 0.298 | 0.0894 | Yes |
| 13 | MARCKS |  |  | 96 | 0.295 | 0.0973 | Yes |
| 14 | RPL22 |  |  | 99 | 0.292 | 0.1067 | Yes |
| 15 | TM9SF3 |  |  | 112 | 0.280 | 0.1076 | Yes |
| 16 | RPS11 |  |  | 113 | 0.279 | 0.1181 | Yes |
| 17 | RPS27A |  |  | 125 | 0.273 | 0.1196 | Yes |
| 18 | TOP1 |  |  | 131 | 0.270 | 0.1258 | Yes |
| 19 | RPS25 |  |  | 133 | 0.268 | 0.1351 | Yes |
| 20 | RPL6 |  |  | 134 | 0.267 | 0.1452 | Yes |
| 21 | RPL35A |  |  | 139 | 0.263 | 0.1519 | Yes |
| 22 | LEPROTL1 |  |  | 145 | 0.260 | 0.1577 | Yes |
| 23 | RPS16 |  |  | 158 | 0.251 | 0.1575 | Yes |
| 24 | INIP |  |  | 171 | -0.251 | 0.1574 | Yes |
| 25 | TERF2IP |  |  | 175 | -0.252 | 0.1645 | Yes |
| 26 | RFX3 |  |  | 186 | -0.252 | 0.1660 | Yes |
| 27 | GNAQ |  |  | 201 | -0.253 | 0.1643 | Yes |
| 28 | STAT2 |  |  | 207 | -0.254 | 0.1698 | Yes |
| 29 | RALGAPA1 |  |  | 230 | -0.257 | 0.1618 | Yes |
| 30 | TANK |  |  | 238 | -0.258 | 0.1660 | Yes |
| 31 | KAT7 |  |  | 248 | -0.259 | 0.1685 | Yes |
| 32 | FTL |  |  | 271 | -0.261 | 0.1607 | Yes |
| 33 | CMIP |  |  | 279 | -0.261 | 0.1649 | Yes |
| 34 | TPR |  |  | 284 | -0.262 | 0.1716 | Yes |
| 35 | FUS |  |  | 290 | -0.263 | 0.1775 | Yes |
| 36 | ZEB2 |  |  | 317 | -0.265 | 0.1666 | No |
| 37 | EIF2AK2 |  |  | 340 | -0.267 | 0.1590 | No |
| 38 | FZD3 |  |  | 351 | -0.268 | 0.1610 | No |
| 39 | ACAP2 |  |  | 367 | -0.270 | 0.1592 | No |
| 40 | SRGAP2B |  |  | 372 | -0.271 | 0.1662 | No |
| 41 | INTS10 |  |  | 409 | -0.274 | 0.1476 | No |
| 42 | BNIP3L |  |  | 431 | -0.277 | 0.1411 | No |
| 43 | DLX1 |  |  | 490 | -0.284 | 0.1052 | No |
| 44 | ANKRD36C |  |  | 501 | -0.285 | 0.1079 | No |
| 45 | SNRNP40 |  |  | 502 | -0.285 | 0.1186 | No |
| 46 | ATP2C1 |  |  | 505 | -0.286 | 0.1278 | No |
| 47 | ZDHHC21 |  |  | 506 | -0.286 | 0.1386 | No |
| 48 | MPRIP |  |  | 577 | -0.296 | 0.0935 | No |
| 49 | BTG3 |  |  | 581 | -0.296 | 0.1022 | No |
| 50 | HERC2 |  |  | 591 | -0.297 | 0.1062 | No |
| 51 | CCDC93 |  |  | 610 | -0.303 | 0.1032 | No |
| 52 | ACADM |  |  | 636 | -0.308 | 0.0947 | No |
| 53 | SCAPER |  |  | 673 | -0.314 | 0.0776 | No |
| 54 | GCC2 |  |  | 712 | -0.321 | 0.0591 | No |
| 55 | DPYSL3 |  |  | 722 | -0.323 | 0.0641 | No |
| 56 | ST8SIA5 |  |  | 737 | -0.327 | 0.0652 | No |
| 57 | PPP1R12A |  |  | 741 | -0.329 | 0.0752 | No |
| 58 | TMSB4X |  |  | 742 | -0.329 | 0.0876 | No |
| 59 | PTPRA |  |  | 772 | -0.337 | 0.0770 | No |
| 60 | HELZ |  |  | 825 | -0.351 | 0.0484 | No |
| 61 | CASK |  |  | 832 | -0.353 | 0.0569 | No |
| 62 | ARFGEF1 |  |  | 845 | -0.356 | 0.0607 | No |
| 63 | RND3 |  |  | 853 | -0.358 | 0.0686 | No |
| 64 | TTLL7 |  |  | 878 | -0.365 | 0.0631 | No |
| 65 | MSH2 |  |  | 927 | -0.385 | 0.0390 | No |
| 66 | HAT1 |  |  | 933 | -0.387 | 0.0497 | No |
| 67 | KIF21A |  |  | 938 | -0.389 | 0.0611 | No |
| 68 | AKAP9 |  |  | 959 | -0.398 | 0.0601 | No |
| 69 | MEG3 |  |  | 975 | -0.404 | 0.0633 | No |
| 70 | DCLK2 |  |  | 986 | -0.408 | 0.0706 | No |
| 71 | KIFAP3 |  |  | 988 | -0.409 | 0.0853 | No |
| 72 | PRKAR2B |  |  | 1005 | -0.416 | 0.0881 | No |
| 73 | PLK2 |  |  | 1047 | -0.444 | 0.0719 | No |
| 74 | MPC1 |  |  | 1086 | -0.462 | 0.0588 | No |
| 75 | CADM1 |  |  | 1103 | -0.469 | 0.0637 | No |
| 76 | GRIA4 |  |  | 1238 | -0.669 | -0.0189 | No |
| 77 | MALAT1 |  |  | 1289 | -0.884 | -0.0257 | No |
| 78 | HIST1H1E |  |  | 1309 | -1.319 | 0.0088 | No |
Table: GSEA details [plain text format]

  

Fig 2: RICHARDS\_NOWAKOWSKI\_SCIENCE\_2017\_NIN2\_UPREG: Random ES distribution      
 Gene set null distribution of ES for **RICHARDS\_NOWAKOWSKI\_SCIENCE\_2017\_NIN2\_UPREG**
